# Horses associate individual human voices with the valence of past interactions: a behavioural and electrophysiological study

**DOI:** 10.1038/s41598-019-47960-5

**Published:** 2019-08-09

**Authors:** Serenella d’Ingeo, Angelo Quaranta, Marcello Siniscalchi, Mathilde Stomp, Caroline Coste, Charlotte Bagnard, Martine Hausberger, Hugo Cousillas

**Affiliations:** 1 Department of Veterinary Medicine, Section of Animal Physiology and Behaviour, University of Bari “Aldo Moro”, Bari, Italy; 2 Université de Rennes, UMR 6552 −Laboratoire Ethologie Animale et Humaine-EthoS-, CNRS, Université de Caen-Normandie, Station Biologique, 35380 Paimpont, France; 3 CNRS- UMR 6552, − Laboratoire Ethologie Animale et Humaine-EthoS-, Université de Rennes, Université de Caen-Normandie, 263 avenue du Général Leclerc, 35042 Rennes, Cedex France; 4 Université de Rennes, UMR CNRS 6552 –Laboratoire Ethologie Animale et Humaine-EthoS- CNRS, Université de Caen-Normandie, Campus de Beaulieu, 263 avenue du général Leclerc, 35042 Rennes, cedex France

**Keywords:** Electroencephalography - EEG, Perception

## Abstract

Brain lateralization is a phenomenon widely reported in the animal kingdom and sensory laterality has been shown to be an indicator of the appraisal of the stimulus valence by an individual. This can prove a useful tool to investigate how animals perceive intra- or hetero-specific signals. The human-animal relationship provides an interesting framework for testing the impact of the valence of interactions on emotional memories. In the present study, we tested whether horses could associate individual human voices with past positive or negative experiences. Both behavioural and electroencephalographic measures allowed examining laterality patterns in addition to the behavioural reactions. The results show that horses reacted to voices associated with past positive experiences with increased attention/arousal (gamma oscillations in the right hemisphere) and indicators of a positive emotional state (left hemisphere activation and ears held forward), and to those associated with past negative experiences with negative affective states (right hemisphere activation and ears held backwards). The responses were further influenced by the animals’ management conditions (e.g. box or pasture). Overall, these results, associating brain and behaviour analysis, clearly demonstrate that horses’ representation of human voices is modulated by the valence of prior horse-human interactions.

## Introduction

Brain lateralization is a phenomenon widely reported in the animal kingdom^1^. Functional brain asymmetries have been observed in several vertebrate species for various sensory modalities, including the auditory sensory domain^2^. In particular, left hemisphere predominance in processing species-specific calls has been found in non-human primates^3,4^, goats^5^, sea lions^6^, raptors^7^ cats^8^ and dogs^9^. On the contrary, right hemisphere activation was observed in vervet monkeys^10^, Japanese macaques^11^ or European starlings^12,13^. In some species such as Mouse lemurs^14^ and Barbary macaques^15^, no bias has been found for in response to conspecific or heterospecific vocalizations. These findings suggest that hemispheric specializations for processing species-specific vocalizations may vary across species. However, as for human language, for which prosody and emotional content are processed in the right hemisphere, there seems to be a differential processing in the two hemispheres for processing different features with different social (e.g. individual information), functional (e.g. familiar/non familiar) significances (e.g^12,13,16^) or attentional significances^17^.

In fact, recent studies report that emotional vocalizations are processed asymmetrically according to their valence.

In humans, a greater frontal activity within the right hemisphere was observed in response to stimuli with a negative valence, whereas a greater frontal left-hemisphere activity was shown in response to stimuli with a positive valence (see for review^18^). A similar laterality bias has been reported in several animal species. Specifically, right hemisphere dominant activity has been shown in response to intraspecific or heterospecific vocalizations related to a negative valence in cats^8^, monkeys^19,20^, dogs^21^ and horses^22^, supporting the general hypothesis of the right hemisphere main involvement in processing intense emotions and arousing stimuli^23,24^. On the other hand, recent studies on dogs^21^ and horses^22^ found that vocalizations expressing a positive emotion (positive valence) were mainly processed by the left hemisphere, which is specialized in processing stimuli eliciting pro-social and affiliative behaviour^23,25^. Although to date the studies on animals have only reported broad differences between right-left hemisphere activation in response to emotional acoustic stimuli, a recent study, examining the dog brain areas involved in processing acoustic stimuli, identified a specific region in dogs’ auditory cortex, namely the right caudal ectosylvian gyri (cESG), which is sensitive to the emotional valence of vocalization and is strongly activated in response to positive vocalizations of both humans and conspecifics^26^. Future studies are needed to identify brain areas involved in auditory processing in different species of animals.

Animal auditory lateralization has been studied employing different techniques that directly analyse brain hemispheres’ activity (e.g. functional magnetic resonance imaging (fMRI)^26^, positron emission tomography (PET)^3^ and electroencephalography (EEG)^27,28^) or by using the head-turning paradigm, which measures an animal preferential use of one ear/eye to attend to a stimulus (e.g.^9,21^). Despite having produced inconsistent results^29^, the latter has been employed to study auditory laterality in a broad range of mammal species, including horses^22,30^, and its suitability is supported by the recent literature (see for review^31^). The head-orienting paradigm measures the animal’s unconditioned and attentive response of turning its head toward the stimuli presented simultaneously on its two sides or behind it. Given that the auditory and visual nervous fibres cross the midline in mammals’ brain, the direction of the head-turning response indicates the contralateral hemisphere activation for processing the stimulus (i.e. right head turns suggest left hemisphere activation and vice versa)^32^. This paradigm requires the animal to be centrally positioned with respect to the stimulus source. Thus, to ensure the correct positioning of the animal, the experiment is usually run during feeding^9,21^ or merely and when appropriate, while it is attached on a long rope to avoid any attentional bias^17,30,33^. In domestic horses (*Equus caballus*) and dogs (*Canis familiaris*) the head-orienting paradigm has been recently employed to study animals’ processing of human vocalizations^21,22,33^. Specifically in dogs, it has been found a different hemispheric specialization in processing the communicative component of human speech, with the right hemisphere mainly involved in perceiving intonational (suprasegmental) vocal cues and the left hemisphere specialized in processing the meaningful phonemic (segmental) cues^33^. However, an opposite lateralized pattern has been shown in a recent fMRI study^34^, which reports the left hemisphere activation for processing intonationally marked words and right hemisphere advantage for processing meaningful words. These findings suggest that further investigations are needed to clarify this issue. Brain asymmetries have also been demonstrated for the processing of emotional human voices in both dogs and horses. In particular, a right hemisphere preference has been reported for vocalizations expressing negative emotions (e.g. sadness or fear), whereas a left hemisphere bias has been found in response to vocalizations expressing positive emotions (e.g. happiness)^21,22^. These findings suggest that dogs and horses are sensitive to the emotional content of human vocalizations.

Functional asymmetries for emotional processing have also been described in human studies, which employed the EEG technique^18,28^. It has been found that the emotional valence affects brain oscillations: in dogs or cats confronted to audio-visual stimuli showing negative in incongruous interspecies (human-pet) interactions, EEG measures revealed more delta waves in the right hemisphere^28^.

The EEG could provide, therefore, a useful and promising tool for investigating brain functional lateralization and emotional processing in both humans and animals.

To date, what is less known is whether animals are able to associate human voices with particular positive or negative experiences. While anecdotal reports indicate that horses may recognize the voice of persons with whom they experienced positive or negative experiences^35^, there has been no scientific proof yet. Associative learning relating particular human voices with past experiences has recently been demonstrated in newborn piglets which showed high stress levels (i.e. distress calls) when presented with the human voices broadcast while their pregnant mother experienced negative handling^36^. These results indicate the existence of a relationship between acoustic associative memories and emotional experiences in pigs.

Similarly to other vertebrate species (e.g. cats:^37^; dolphins and belugas:^38,39^; sheep:^40^), horses discriminate between familiar and unfamiliar humans using vocal and visual cues^41,42^. Moreover, they form a long-lasting and cross modal representation of humans^41,43,44^. This representation and the associated emotional appraisal are built on the emotional valence of previous interactions, and influences horses’ reactions in subsequent interactions with humans^45,46^. In particular, recent studies reported that horses trained with a positive reinforcement displayed an increased interest toward humans and sought more contact from them; whereas a negative reinforcement elicited an increase in the horses’ emotional state and induced less contacts^47,48^. These observations were confirmed after a long period without contact (6–8 months) and with unknown persons^47^, confirming that a generalization process occurred^45,47,49^. However, contact seeking remained stronger for the familiar trainer using positive reinforcement than for any other persons, revealing further an association between the human individual characteristics and the valence of this past experience^47^.

Nevertheless, evidence about the interplay between acoustic associative memories and prior emotional experiences, and its influence on the current representation horses build of particular humans remains largely unknown. In the present study, we hypothesized that horses may associate particular human voices with the valence of earlier experiences.

In order to test this hypothesis, we submitted, for seven consecutive days, twenty-one horses to a positive (i.e. food) or negative (i.e. food soaked with vinegar, inducing frustration^50^) experience while hearing one of two different human voices. A particular pair of voices (one associated with the positive experience and the other with the negative experience) was used for each horse. During the interactions (bucket presented and given) the human voices were broadcast continuously through a small loudspeaker worn by the silent experimenter (one per each experience type). The acoustic stimuli consisted of a human voice reading a standardized text (from Tallet *et al*.^36^, see Supplementary information for the acoustic stimuli text). After a “training phase” of 7 days, aiming at creating acoustic associative memories with the valence of the experiences with humans, a test condition was performed, where the two human voices were broadcast to each subject, separately. Horses’ behavioural reactions and lateralization patterns were assessed. Horses’ lateralized responses to the acoustic stimuli were tested by using both the head-turning paradigm and the EEG technique. Moreover, since management practices may influence horses’ emotionality and reactions to humans (e.g.^45,51,52^), we investigated the differences in the individuals’ perception of human voices in two differently managed populations.

## Results

### Behavioural responses

Overall, horses responded with changes of behaviour, i.e. head turning, with the same frequency (55.6% for the voice associated with the positive experience V+ and 77.8% for the voices associated with the negative experience V−; P > 0.05; McNemar test) and the same latency (V+: 4.46 ± 2.71 s; V−: 3.71 ± 2.44 s, mean ± S.D.; P > 0.05; t-test paired samples) to differently valenced voice playbacks.

**Figure 1:**
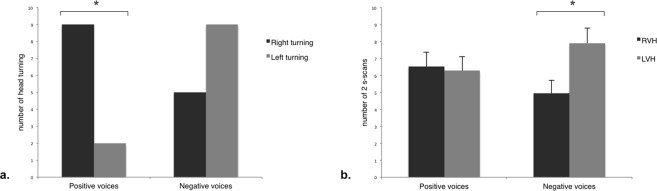
Head-orienting response and visual laterality at total population level in response to the broadcast of V+ (“positive” voices) and V− (“negative” voices). (**a**) Total number of right and left head-turning (One-sample Wilcoxon signed rank test); (**b**) Number of scans in which the horses, when released, had the loudspeaker in the right (RVH) and left (LVH) visual hemifield, according to the stimulus valence (means and S.E.M. are shown; Wilcoxon signed rank test). *P < 0.05.

However, the two types of voices were clearly differentiated as, when the horses were handled there were more right head turning for V+ (N = 15, Z = 54.00, P = 0.035; One-sample Wilcoxon signed ranks test), whereas there was no laterality bias for V− (N = 17, Z = 37.50, P = 0.285) (Fig. 1a). When the horses were released, they spent most time with the loudspeaker in a monocular rather than a binocular field (V+: N = 21, Z = 20.00, P = 0.001; V−: N = 21, Z = 0.00, P = 0.00; Wilcoxon signed rank test). In particular, they spent more time with the loudspeaker on their left side while V− was being broadcast (N = 21, Z = 173.50, P = 0.044), whereas no lateral biases were observed for V+ (P > 0.05) (Fig. 1b).

Moreover, when held, the horses spent most time with the ears forwards during the playback of V+ (N = 21, Z = 22.00, P = 0.029; Wilcoxon signed rank test); whereas they spent more time with their ears backwards when V− was being broadcast than when it was V+ (N = 21, Z = 106.00, P = 0.048).

Despite the fact that the horses showed visual attention towards the voices, as shown by head-turning responses, they generally remained quiet and there was no significant difference overall in the other behaviours expressed towards the two categories of voices (Frustration and Vigilance: P > 0.05; t-test paired samples; Visual attention: P > 0.05; Wilcoxon signed rank test). 60% of the horses showed a frustration-related behaviour at least once and 69% expressed vigilance at least once, but when the “negative behaviours” (i.e. Vigilance and Frustration categories) were pooled, no differences were found between V+ and V− (P > 0.05).

Anecdotally, 3 horses pulled the rope to turn their head and body toward the source of V+, or even approached it (one of them with a nicker, see Supplementary Video S2), and 7 pulled the rope to move forward for V−, increasing their distance from the sound source (see Supplementary Video S1). Finally, although the time spent with the loudspeaker in a binocular field was higher for the first than for the second voice heard (N = 21, Z = 0.00, P = 0.008; Wilcoxon signed rank test), no effect of the order of voices presentation to the horses was observed for all the other analysed parameters (P > 0.05).

When comparing riding centre and leisure horses, it appeared that the perception of the experimental situation varied only slightly according to the population. There was no difference according to the population neither in the number of responses (i.e. head-turning) to the voices (V+: L = 66.7%, RC = 44.4%; V−: L = 77.8%, RC = 77.8%) (P > 0.05; McNemar test), nor in the time spent with the loudspeaker on one side whatever the voice valence (P > 0.05; Mann-Whitney test). There were only minor differences according to the population: 1) the riding centre horses, but not the leisure horses, turned more the head to the right in response to V+ (N = 6, Z = 10.00, P = 0.046; One-sample Wilcoxon signed ranks test); 2) the riding centre horses spent more time with the ears forward during the playback of V+ than V− (N = 10, Z = 2.50, P = 0.028), and the leisure horses spent more time with the ears backwards for V− than for V+ (N = 21, Z = 3.10, P = 0.002); 3) the horses living in the riding centre showed more frustration-related behaviour than the leisure horses in response to V− (N = 21, U = 95.50, P = 0.003; Mann-Whitney test). Therefore, the discrimination of voices and their association with the past experiences were present in both populations but the riding centre horses showed more sensitivity to the associated valence.

### Electroencephalography

Electroencephalographic recordings (EEG) of seventeen horses were taken during a baseline period (8 s before the stimuli onset) and during the voices broadcast (8 s after the stimuli onset). Thus, the EEG power profiles computed for the baseline period and the stimuli broadcast could then be compared and analysed.

**Figure 2:**
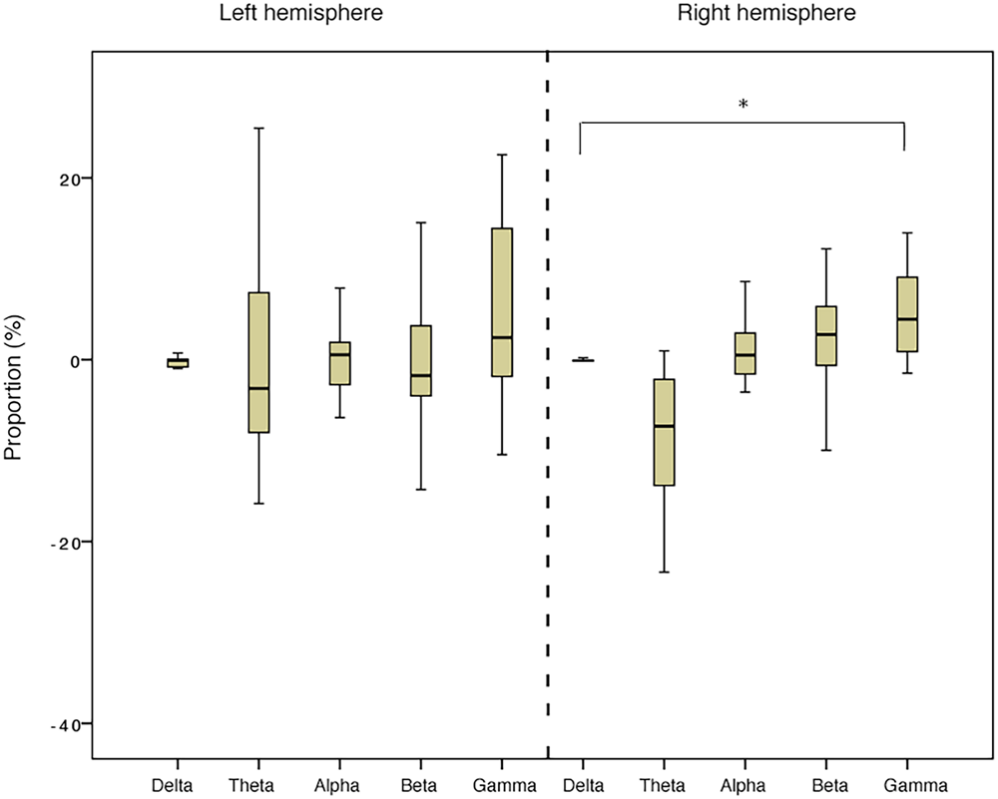
Wave median proportions of the total population in the right and in the left hemisphere in response to the “positive” voices V+ (Friedman test). *P < 0.05 (explanations can be found in the main text).

Results for the EEG confirmed that, at the total population level, horses reacted differently to the two acoustic stimuli presented. Specifically, there was a statistically significant difference in the relative power of the wave frequencies in response to V+ (N = 14, χ2 (9) = 20.96, P = 0.013; Friedman test), with a clear increase of gamma waves in the right hemisphere, significantly more frequent than delta frequency bands (N = 16, Z = −2.22, P = 0.026; Wilcoxon signed rank test) (Fig. 2). No statistically significant differences in the wave proportions were found in the left hemisphere (P > 0.05; Wilcoxon signed rank test) and no laterality biases were observed for V− (P > 0.05; Friedman test). Moreover, the order of voices presentation to the horses had no effect on all the tested parameters (P > 0.05), with the exception of the alpha frequency bands in the left hemisphere, which were higher for the first then the second voice heard (N = 15, Z = 24.00, P = 0.041; Wilcoxon signed rank test).

**Figure 3:**
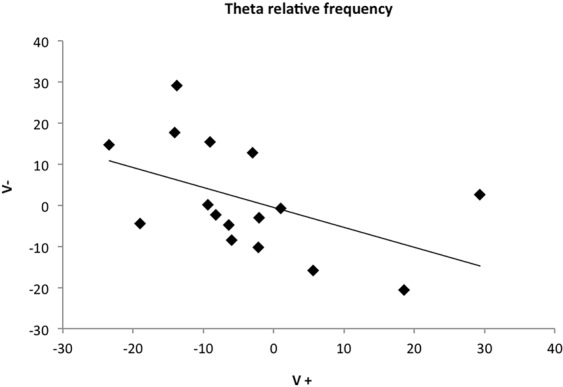
Theta wave relative frequency in % of the power profile (right hemisphere): correlation between data obtained for each horse during the playback of V+ and V− respectively.

Data analysis showed also that the EEG profiles were opposite for the two types of voices, with a negative and statistically significant correlation for gamma (V+ and V−: N = 16, r = −0.588, P = 0.017) and theta (V+ and V−: N = 16, r = −0.538, P = 0.031; Spearman correlations; Fig. 3) waves in the right hemisphere. However, both hemispheres seemed to be equally activated by the broadcast of both types of voices, as a positive correlation between alpha waves in the left hemisphere was found (N = 15, r = 0.549, P = 0.034).

## Discussion

This study confirms that horses do discriminate human individual voices but also reveals that they have a memory of the valence of past experiences with these voices. Specifically, their reaction to the broadcast of human voices is modulated by the valence (positive or negative) of the prior interactions with humans. Human voices associated with previous positive experiences elicited a positive response in the horses and promoted their attention, whereas human voices associated with past negative experiences produced a negative affective state in the tested subjects’. Behavioural lateralization patterns, moreover, showed that horses had indeed associated each voice with a different emotional experience. In particular, horses reacted to the voices associated with the positive interactions by turning their head to the right side. Given that the auditory information is processed in the hemisphere contralateral to the direction of the head-turning response^30^, the right bias here found suggests the left hemisphere advantage for processing the “positive” voices. This finding is consistent with the left hemisphere role found in several vertebrates for the expression of pro-social and approaching behaviour and with the control of the response to stimuli regarded as positive^23^. Moreover, horses displayed a positive and attentive posture, namely ears forwards, in response to the “positive” voices, which indicates a positive perception of a situation or an interaction^45,53^. These findings, together with some individuals’ tendency to approach the sound source (i.e. the loudspeaker), suggest that the horses associated the positive valence of the experience with the human voices broadcast during the human-horse interactions, and subsequently perceived these voices as positive stimuli. The influence of interactions on the subsequent perception of human voices has also been recently described in piglets, which displayed more stress behaviours during the broadcast of the human voices heard prenatally while their mother experienced a negative emotional state^36^.

The analysis of horses’ electroencephalographic (EEG) profiles revealed a clear prevalence of the gamma waves in the right hemisphere in response to the “positive” voices. The increase of gamma wave proportions in the right hemisphere has been found to be associated with attention in horses^27^ and with expectancy in a cognitive task in humans^54^. The involvement of the right hemisphere in attentional processing has been discussed lately for different species as well^17^. Thus, the prevalence of gamma waves in the right hemisphere described here could be explained by the animals’ expectancy to receive food when hearing the “positive” voices. In the light of the EEG and behavioural results, we could hypothesize that, after the initial positive perception of the “positive” voices (left hemisphere activity and forward ears’ position), the memory of the positive interactions with humans could have elicited an increase in animals’ attentional state toward human voices (increased gamma power in the right hemisphere), producing positive expectation to obtain food. Therefore, the left hemisphere first involvement followed by the right hemisphere activation could indicate a different cognitive process for the perception and then integration of the acoustic “positive” signals.

On the other hand, the horses used preferentially their left eye to investigate the sound source (i.e. the loudspeaker) when hearing the human voices broadcast during the negative interactions, suggesting a right hemisphere main involvement in processing such voices (80–90% of the optic fibres decussation in horses’ brain^55^). This finding is consistent with the right hemisphere specializations for the perception and the expression of intense/negative emotions and the control of rapid responses previously described for several vertebrate species^1,56^. Specifically in horses, the right hemisphere activation has been reported in the visual analysis of potentially fear-inducing stimuli (e.g. novel objects^57^ or humans^58^) and clearly negative stimuli (i.e. white coat worn by the veterinarian^59^). Thus, the horses’ left-eye preferential use (right hemisphere) to investigate the source of the human voices (i.e. the loudspeaker) can be related to the animal emotional association with the frustrating/negative earlier experience with this same voice^1^. Moreover, the horses directed their ears backwards in response to the “negative” voices, indicating discomfort and a negative perception of a situation or an interaction^45,53^.

Horses’ different perception of the valence of human voices was further confirmed by EEG profile analysis, which revealed a negative correlation between the “positive” and “negative” voices of gamma and theta relative power in the right hemisphere. In other words, the voices with an opposite valence produced opposite changes in gamma and theta relative power. These findings are consistent with EEG studies on humans’ domain, showing a different hemisphere involvement in processing emotions with opposite emotional valence (i.e. positive and negative). In particular, it has been reported a right hemisphere activation in response to negative stimuli and a left hemisphere activation in response to positive stimuli^18,28^. Oscillation of theta and gamma bands are involved in several aspects of memory, including the information encoding of memory-relevant objects^60^, as well as the consolidation and the retrieval of stored memories^61^. Specifically, a link between theta activity and emotional states/regulation has been reported both in humans and in horses^28,62^. More recently, it has been found that theta oscillations are synchronized within the right hemisphere when humans attend to a negative emotional stimulus more than a positive one^28^. Regarding gamma bands, oscillations in its power are generally associated with high-level mental activities, such as emotions^54^ and stimuli related sensory/cognitive functions^63^. Although the opposite changes of gamma and theta bands suggest a different and opposite pattern of brain processing of the “positive” and “negative” voices, the specific role of each wave in the emotional processing and emotional state in horses still needs further investigations. On the contrary, although the alpha oscillations were higher for the first voice heard, a positive correlation of alpha bands activity between the “positive” and “negative” voices in the left hemisphere was found. This relationship can be explained by the alpha bands involvement in memory demands and in mental representation of objects and events, which could have occurred for both the acoustic stimuli^62^.

Overall, both behavioural and electrophysiological results demonstrate that horses not only associate human voices with the valence of previous experiences with humans, but they also recall the valence of such experiences when hearing human voices. These findings support anecdotal reports of long-term memories of past experiences with humans^35^. They also confirm the evidence that horses build representations of humans^41,44^ that are influenced by daily interactions^46,49^ or training modalities^47,48^. Therefore, the valence of previous interactions can affect horses future attitude towards humans and their behaviour^45^. Our results showed that positive interactions produce positive expectations and a positive attitude to interact with humans, promoting animal attention and approaching behaviour^64^. On the contrary, negative experiences cause negative affective states and consequently negative expectations about the forthcoming interactions.

Furthermore, our results demonstrate that management conditions modulate horses’ perception of the valence of their interactions with humans and influence their behaviour during future interactions. In particular, riding centre horses, which lived in restricted conditions, appeared to be more sensitive than leisure horses to the different valences of the prior experiences associated with human voices. The acoustic stimuli, indeed, induced two opposite reactions in riding centre horses, which consistently turned their head to the right (left hemisphere activation) and held their ears forwards in response to the “positive” voices; whereas they displayed more frustration-related behaviour when hearing the voices associated with the negative interactions. The higher level of frustration showed in response to these voices by riding centre horses compared to the leisure horses, which also perceived these stimuli as negative (backwards ears’ position^45^), can be explained by their general higher sensibility to stress due to their more restricted management conditions^51,65^, which includes also feeding. The leisure horses had grass or hay ad libitum and may therefore have been less sensitive to the bringing of additional food or to the frustration to be unable to eat the “spoiled” food. This shows further that horses do memorize the association between the valence (and intensity) of the human-horse interaction and human characteristics.

This study is the first that combines behavioural observations with electroencephalography in fully awake animals to study horses’ emotional memory of past interactions with humans. It provides new insights into the mechanisms involved in the role of the valence of interactions on sensory memories and has also important implications for the role of humans in the cognition and welfare of domestic/captive animals.

## Methods

### Ethical statement

The experiment was carried out in accordance with directive 2010/63/EU of the European Parliament and the French law relative to the protection of the animal used in scientific experiment (Décret n°2013-118 13 février 2013; Article R. 21488). This experiment only included behavioural observations and non-invasive interactions with the horses and consequently did not require an authorization to experiment.

### Subjects

The study was carried out in Brittany (France) in June and July 2017 on twenty-one horses of various breeds, aged 2 to 22 years (10.90 ± 5.48; mean ± S.D.). They belonged to two differently managed populations. The first population (6 mares and 4 geldings, aged 8–17 years, 11.90 ± 3.03; mean ± S.D.) belonged to a riding centre (RC) and lived in restricted conditions: single 3.4 × 3.3 m stalls, limited access to roughage (5–7 kg once a day) and constrained riding techniques (riding lessons, English riding style, 3–4 hours a day^66^). The second population (leisure horses (L): 6 mares, 3 stallions and 2 geldings, aged 2–22 years, 10.00 ± 7.07) belonged to the University of Rennes 1 or to a private owner and lived in naturalistic conditions (6 stable groups of 2–4 individuals in 1–2 ha pasture with grass or hay ad libitum). These horses were occasionally used for leisure riding (i.e. low hands and long reins) and had daily interactions with humans (visits and occasional food).

### Acoustic stimuli

Voices of twenty-two women, aged 21 to 62 years (34.29 ± 11.22; mean ± S.D.), were recorded in a soundproof chamber with a 44100 Hz sampling frequency using GoldWave® v5.70 software. Subjects had to read a standard text previously used by Tallet *et al*.^36^ (see Supplementary information), containing all the French phonemes and without any emotional connotations. Each reading lasting 17 s was repeated three times with a 4 s interval. Hence, acoustic stimuli of 1 min (60.00 ± 1.28) were obtained. The recordings were then equalized and their amplitude homogenized using GoldWave® v5.70 software.

A pair of voices was randomly assigned to one horse of each group (hence one horse in each population received the same couple of voices, but voices used in the same population were all different) and each voice was associated with a positive or a negative experience with an experimenter for one horse. This prevented pseudoreplication.

### Training

For 7 consecutive days horses had two 10-minute sessions of individual interactions in their home environment (box or pasture) with two unknown female experimenters (trainers), each of them associated with a positive and a negative experience and wore a same blue coat. They remained silent but wore a loudspeaker (JBL-GO®) hanging on their chest and connected to a mp3 player (DJIX M340FM®) broadcasting a voice (one same voice per experimenter per horse) during the bucket presentation to the horse. Each bucket was filled with food for the “positive” experimenter, or with food soaked in red vinegar (unpalatable thus inducing frustration^50^) for the “negative” experimenter. Thus, during daily interactions with the two experimenters (mean interval between the interactions with the two experimenters 4 ± 2 min), each horse was presented successively with both the negative and the positive experience (the order changed randomly over days and between horses) and the two corresponding human voices.

The acoustic stimuli broadcast started when the experimenter was frontally positioned at a distance of about 50 cm from the horse, and placed the bucket under the horse’s head. The average loudness of the sound measured from the horse position was 60 dB. The succession of experimenters between days for each horse and between subjects for different days varied randomly. The riding centre horses were tested in the morning before the first meal was provided, so that they were motivated, whereas the leisure horses were trained later in the morning, since they usually received no such meal. Riding centre horses were presented with the human voice and the bucket one at a time and individually in their own box. For the leisure horses, the other group members were retained in another enclosed part of the pasture during training (and testing) of the target individual.

## Test

### Experimental setup

The experiment was carried out in a familiar environment, in particular in the covered arena of the riding centre and in the pasture where the leisure horses lived. A loudspeaker (Sony SRS77G®) connected to an mp3 player (DJIX M340FM®) was placed centrally and at 10 m behind the horse and out of its view, according to the paradigm used in Basile *et al*.^30^. The average loudness of the acoustic stimuli measured from the horses’ head position was 60 dB. Two digital video-cameras, placed centrally in front and behind the horse, were used to record continuously horses’ behaviour during the test. One experimenter (Sd or HC) controlled the stimuli broadcasting from a designated position, located in front of the horse at a distance of about 2 m and centrally positioned, to avoid any bias in horses’ lateralized response. Moreover, they stand motionless during the playbacks and avoid interacting with the tested subject (gazing at the computer to control stimuli broadcasting and check the electroencephalographic recordings).

### Procedure

The test took place the day after the end of the training. The two voices, previously associated with the positive and the negative interactions, were presented consecutively to each horse, in a random order between subjects. The test was carried out without the presence of the two trainers and food. An unfamiliar experimenter (MSt or MH) handled the horse, positioning herself centrally and in front of the animal, to avoid any bias in the head-turning response. Once the position of the horse’s head was central and symmetric with respect to the loudspeaker, the acoustic stimulus was broadcast. The horse was halter-restrained during the first text reading (first 17 s)^30^ and was then released in order to see potential approaches to the sound source.

After releasing the horse, the handler joined the other experimenters (Sd or HC) staying still till the end of the stimulus presentation (see Supplementary Videos S1 and S2).

### Data analysis

Horses’ behaviour was video-recorded during the whole test and then analysed using continuous focal animal sampling by one trained observer (Sd), who was blind to the voice valence.

Asymmetries in the head-turning response and in the visual hemifield used for attending to the loudspeaker were analysed. The latency between the stimuli onset and the head-orienting response was computed and a threshold of 7.5 s from the sound onset was set up (see Supplementary Fig. S1 and supplementary information for the criteria selection). Moreover, the time spent by the horse, when released, with the loudspeaker in its right or left visual hemifield was assessed using instantaneous scan sampling (every 2 s). Ears’ positions were recorded using instantaneous scan sampling (every 2 s) while the horses were handled (the first 17 s of the playback). Two main different positions were considered: ears directed backwards, occurring mostly in agonistic and “negative” interactions or discomfort, and ears directed forward, which indicates attention or a positive perception of a situation or interaction^45^. The total number of scans in which both ears were directed forward and backwards was computed for each subject. Other positions, such as asymmetrical or sideward positions, which were rarer and more ambiguous (no effect of treatment in pilot analyses), were not further analysed.

Three other behavioural categories were considered: *frustration* (chewing, lips movements, head shaking, pawing, yawning), *vigilance* (standing still, head/neck up, eyes open and alert, tail raised, neck arched), *visual attention directed toward the loudspeaker* (glance the loudspeaker <1 s, gaze the loudspeaker >1 s, head and eyes directed toward the loudspeaker), *approaching behaviour* (decrease the distance from the loudspeaker <9 m)^53^. The frequency of each behaviour was analysed.

### Electroencephalographic recordings

Seventeen out of the 21 horses (8 riding centre and 9 leisure horses) could be used for electroencephalographic recordings (EEG; not allowed by owners for 4 horses). For a week before the beginning of the test, horses were trained daily to wear a non-invasive EEG headset recently developed by Cousillas *et al*.^67^ (patent # R23701WO) that allows EEG recordings in free moving animals. The headset is made up of 4 electrodes positioned on the frontal and parietal bones (two for each side of the head) and of one ground electrode placed on the back of the left ear. It is also composed of a telemetric EEG recorder made by RF-TRACK (Cesson-Sevigne, France) and an amplifier based on Texas Instruments integrated circuit ADS1294 connected to a Bluetooth transmitter.

Before the beginning of the test, the helmet was positioned on the horses’ head and recordings started as soon as the horse was in the designated position. For the analysis, the 8 s before (baseline) and the 8 s following stimulus onset were analysed and compared. This time window was chosen with regards to behavioural response latencies (see above).

For the data analysis, the large artefacts due to the animals’ body movements were removed using a smoothly Savitzy Golay function integrated in a homemade software made with Python 3.6.4 environment. The device sampling-rate was 250 Hz and the signal was recorded using EEG software “EEGReplay4.3” developed by RF-TRACK. This software calculated the proportion of the five main brain waves in the mean power: delta (δ: 0–4 Hz), theta (θ: 4–8 Hz), alpha (α: 8–12 Hz), beta (β: 12–30 Hz) and gamma (γ: > 30 Hz)^68^. The median of the percentage values of each frequency band recorded during the 8 s before and 8 s after the stimuli onset was computed, reflecting the median percentage value of each wave type during basal activity (baseline) and in response to the acoustic stimuli for each horse. The EEG power profiles recorded during the baseline period and the stimuli broadcast were then compared for each tested subject.

### Statistical analyses

The statistical analysis was performed using SPSS software. Data distribution was tested using Shapiro–Wilk test. According to data distribution, Wilcoxon signed rank tests and paired samples t-test were used to test differences between the “positive” and “negative” voices for several parameters: visual laterality, animals’ behaviour and ears’ positions. McNemar test was used to test differences in the frequency of head turning response between the “positive” and “negative” voices. Differences between the two populations of horses in their response to the acoustic stimuli were assessed via Mann-Whitney test. Asymmetries at a group-level in the head-turning response were assessed via One-Sample Wilcoxon Signed Ranks Test, to report significant deviation from zero. Differences in the brain waves proportions between the right and in the left hemisphere were tested using a Friedman test. Moreover, differences in the EEG relative median percentages of different wave frequencies in each hemisphere and between the two hemispheres were analysed by Wilcoxon signed rank tests. In addition, Spearman correlations were used to measure the association between the proportions of two waves frequency bands and the direction of their relationship. For both the behavioural and the EEG data, the order effect of the stimuli presentation was tested via Wilcoxon signed rank tests. Results were considered statistically significant for P < 0.05.

## Supplementary information

Supplementary information

Supplementary Video 1

Supplementary Video 2

## Data Availability

The datasets generated during and/or analysed during the current study are available from the corresponding author on reasonable request.
