# Supplementary material for: Horses associate individual human voices with the valence of past interactions: a behavioural and electrophysiological study
Source: Sci Rep. 2019 Aug 9;9:11568. doi: 10.1038/s41598-019-47960-5 (PMC6689011; doi:10.1038/s41598-019-47960-5)
Supplement: Supplementary file 1 — Supplementary information [file 41598_2019_47960_MOESM1_ESM.pdf]

Supplementary Information

Title of manuscript:

Horses associate individual human voices with the valence of past interactions: a behavioural and electrophysiological study

Authors:

Serenella d'Ingeo[1,2*], Angelo Quaranta[1], Marcello Siniscalchi[1], Mathilde Stomp[2], Caroline Coste[2], Charlotte Bagnard[2], Martine Hausberger[3†], Hugo Cousillas[4†]

[1]Department of Veterinary Medicine, Section of Animal Physiology and Behaviour, University of Bari "Aldo Moro", Bari, Italy.
[2]Université de Rennes, UMR 6552 –Laboratoire Ethologie Animale et Humaine-EthoS-, CNRS, Université de Caen- Normandie, Station Biologique, 35380, Paimpont, France.
[3]CNRS- UMR 6552, – Laboratoire Ethologie Animale et Humaine-EthoS-, Université de Rennes, Université de Caen-Normandie, 263 avenue du Général Leclerc, 35042, Rennes, Cedex, France
[4]Université de Rennes, UMR CNRS 6552 –Laboratoire Ethologie Animale et Humaine-EthoS- CNRS, Université de Caen-Normandie, Campus de Beaulieu, 263 avenue du général Leclerc, 35042, Rennes, cedex, France
† These authors contributed equally to this work

* Corresponding author

Correspondence to serenella.dingeo@uniba.it

Acoustic stimuli text

Voices of volunteers were recorded while reading a text previously used by Tallet et al. [36], containing all the phonemes of the French language and with any emotional connotation: "*Petit Louis, les yeux ouverts, rêvait dans son lit bleu. Le jour des vacances était arrivé. Il sentait l'odeur du bon pain chaud et du chocolat que maman préparait. Papa et lui iraient à la gare chercher son cousin. Ils feraient du camping à la campagne. Louis n'aurait plus peur des ruades de l'âne brun*."

Latency threshold for head-turning response

The latency time between the stimuli onset and the head-orienting response was computed. The latency threshold was set up following the method described by Basile et al. [30], based on the visual inspection of the frequency distribution of the head turning response (see Supplementary Fig. 1). The head-turning response was considered valid when it occurred within 7.5 s from the sound onset. Three different responses were then considered: head turning to the right side, left head-turning response and no response (if the horse did not turn its head within 7.5 s after the sound onset).

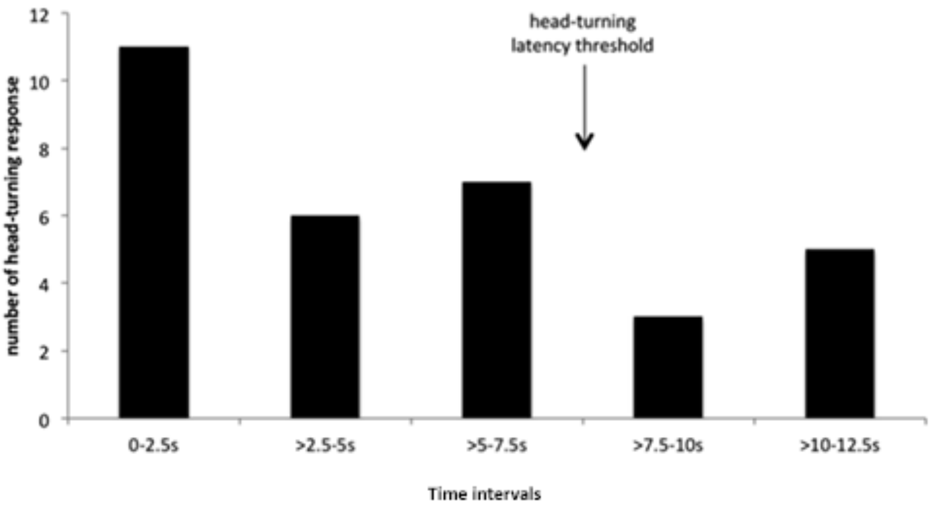

Supplementary Figure 1. Number of head-turning responses according to the time after the stimuli onset.
